# Monounsaturated Fatty Acids Are Substrates for Aldehyde Generation in Tellurite-Exposed *Escherichia coli*

**DOI:** 10.1155/2013/563756

**Published:** 2013-08-07

**Authors:** Gonzalo A. Pradenas, Waldo A. Díaz-Vásquez, José M. Pérez-Donoso, Claudio C. Vásquez

**Affiliations:** ^1^Laboratorio de Microbiología Molecular, Departamento de Biología, Facultad de Química y Biología, Universidad de Santiago de Chile, Santiago de Chile, Chile; ^2^Laboratorio de Microbiología y Bionanotecnología, Facultad de Ciencias Químicas y Farmacéuticas, Universidad de Chile, Santiago de Chile, Chile; ^3^Laboratorio de Bionanotecnología y Microbiología, Centro de Bioinformática y Biología Integrativa (CBIB), Facultad de Ciencias Biológicas, Universidad Andres Bello, Santiago de Chile, Chile

## Abstract

Reactive oxygen species (ROS) damage macromolecules and cellular components in nearly all kinds of cells and often generate toxic intracellular byproducts. In this work, aldehyde generation derived from the *Escherichia coli* membrane oxidation as well as membrane fatty acid profiles, protein oxidation, and bacterial resistance to oxidative stress elicitors was evaluated. Studies included wild-type cells as well as cells exhibiting a modulated monounsaturated fatty acid (MUFA) ratio. The hydroxyaldehyde 4-hydroxy 2-nonenal was found to be most likely produced by *E. coli*, whose levels are dependent upon exposure to oxidative stress elicitors. Aldehyde amounts and markers of oxidative damage decreased upon exposure to *E. coli* containing low MUFA ratios, which was paralleled by a concomitant increase in resistance to ROS-generating compounds. MUFAs ratio, lipid peroxidation, and aldehyde generation were found to be directly related; that is, the lower the MUFAs ratio, the lower the peroxide and aldehyde generation levels. These results provide additional evidence about MUFAs being targets for membrane lipid oxidation and their relevance in aldehyde generation.

## 1. Introduction

Oxidative damage is a widespread phenomenon affecting nearly all cell types which harm several macromolecules including DNA, proteins, and fatty acids [1, 2]. This kind of damage results largely from reactive oxygen species (ROS) generation that can be produced in a number of ways, from exposition to metal/metalloids [3–5] and to their normal formation during respiration in aerobes [6] and as part of the cellular immune response in humans [7], among others. Since the overall ROS effect on bacteria is not fully understood, it is tempting to focus on unexplored approaches. In this context, the oxidation of particular cell components—in addition to the direct damage they suffer—results in the formation of several toxic byproducts such as ROS-derived aldehydes. In eukaryotic cells, aldehyde generation comes mainly from the oxidation of membrane polyunsaturated fatty acids (PUFAs); initially formed lipid peroxides can then be decomposed to different reactive aldehydes that in turn cause damage to different cellular components [8].

Although oxidative stress effects on *Escherichia coli* have been widely analyzed [6], the formation of ROS-derived aldehydes has not been particularly considered in this bacterium. The little available evidence comes from the *E*. *coli* aldehyde reductase YqhD, an enzyme whose activity confers resistance to several oxidative stress elicitors by decreasing aldehyde-mediated cell damage [5]. Since PUFAs are absent from the *E*. *coli *membrane [9], it seems unlikely that these molecules could represent the aldehyde source in this bacterium. In contrast, monounsaturated fatty acids (MUFAs) do exist in *E. coli* [9] which seem to be good candidates for aldehyde production as they can be oxidized to form lipid peroxides [10–12]. This assumption is supported by observations pointing out that the *E*. *coli* resistance to several oxidative stress elicitors is dependent on membrane MUFA composition [13]. In this work, aldehyde generation was found to occur in *E*. *coli* under toxicant-induced oxidative stress conditions which are dependent on the membrane MUFA ratio. In addition, it also results in modulating lipid-derived peroxide levels, oxidized proteins, and *E*. *coli* resistance to oxidative stress elicitors.

## 2. Materials and Methods

### 2.1. Plasmid Construction and Culture Conditions

Plasmids p*fabA *and p*cfa *were generated by ligating PCR-amplified *E. coli fabA *[ATGGTAGATAAACGCGAATCC (forward), GAAGGCAGACGTATCCTGGA (reverse)] and* cfa* [ATGAGTTCATCGTGTATAGAAGAAG (forward), GCGAGCCACTCGAAGGCCGT (reverse)] genes to pBAD TOPO expression vector (Invitrogen) and introducing them into *E. coli *Top 10 by electroporation. Correct gene orientation and insertion was confirmed by *Nco*I digestion and PCR amplification. Then pBAD (control), p*fabA,* and p*cfa* plasmids were introduced independently into *E. coli *BW25113 Δ*yqhD*. Cells were routinely grown at 37°C in ampicillin (100 *μ*g/mL) and kanamycin (30 *μ*g/mL) amended Vogel-Bonner (VB) minimal medium [14].

### 2.2. Aldehyde Content Determination

Cell cultures (500 mL, OD_600_~0.3) were induced with 0.02% arabinose for 2 h and exposed to 0.25 *μ*M TeO_3_^2−^, 0.3 mM H_2_O_2_, or 0.015 mM *tert*-butyl hydroperoxide (TBH) for 30 min. After washing with 10 mM potassium phosphate buffer pH 7.0 (buffer A), cells were suspended in 500 *μ*L of buffer A, mixed with 500 *μ*L of cyclohexanedione reagent [(ammonium acetate (10 g), acetic acid (10 mL), and 1,3-cyclohexanedione (0.25 g) in 100 mL of mili-Q water)], and incubated at 60°C for 1 h. After centrifuging at 5000 × g for 10 min, the pellet was discarded, and aldehyde adducts were concentrated in a Sep-pak C18 (Waters) column. After washing with 3 mL of mili-Q water, adducts were eluted with 1 mL of HPLC-grade methanol. The alcohol was evaporated in a SpeedVac apparatus and the sample washed with 500 *μ*L of chloroform, centrifuged to precipitate any remaining salt, and evaporated. Adducts were suspended in 50 *μ*L of methanol : H_2_O (1 : 1), and 20 *μ*L of the mixture was injected in a C18 HPLC column (Agilent) along with 2.5 *μ*moles of octanal as internal standard. The HPLC program was 0–9 min methanol : H_2_O (3 : 7), 9–16 min isocratic flux of tetrahydrofuran : H_2_O (THF : H_2_O, 26 : 74), 16–21 min isocratic flux THF : H_2_O (4 : 6), and 21–25 min isocratic flux THF 100%. The flow was set at 2 mL/min and the fluorescence of the aldehyde adducts (380 nm excitation, 446 nm emission) was detected.

### 2.3. Determination of the Minimal Inhibitory Concentration (MIC)

Cells from overnight cultures were diluted 100-fold with fresh medium, grown to OD_600_~0.3, and 10 *μ*L aliquots were inoculated into 96-well plates containing serial dilutions of the particular toxicant in 150 *μ*L of antibiotic-amended medium and plates were incubated for 48 h at 37°C with gentle shaking. ROS elicitors tested included the superoxide-generating compound tellurite [5], the lipid-peroxidating agent TBH [15] and hydrogen peroxide [6].

### 2.4. Protein Carbonylation

Protein carbonyl group content was determined as described earlier [16, 17]. Briefly, nucleic acids-free cell extracts (100 *μ*L) from *E. coli *BW25113 Δ*yqhD* exposed to the toxicants were mixed with 4 volumes of 2 M HCl containing 10 mM dinitrophenylhydrazine and incubated for 1 h at room temperature with vortexing every 15 min. Proteins were precipitated with 1 volume of trichloroacetic acid (20%) and pelleted at 10000 ×g for 10 min. After washing 3 times with ethanol : ethyl acetate (1 : 1), the pellet was dissolved with 450 *μ*L of 6 M guanidine hydrochloride containing 50 mM dithiothreitol. Carbonyl content was determined at 370 nm using a molar absorption coefficient of 22,000 M^−1 ^cm^−1^ [18].

### 2.5. Fatty Acid Profile Determination

Bacterial cultures (50 mL) were exposed for 30 min to the toxicants, sedimented, and dehydrated in a SpeedVac equipment. Pellets were suspended in 500 *μ*L of methanolic HCl (5%) and incubated at 80°C for 10 min to produce fatty acid-derived methyl esters (FAMEs). Resulting mixtures were extracted with hexane (500 *μ*L) and evaporated to a volume of 50 *μ*L. Five *μ*L of each sample were injected in a diphenyl-dimethylsiloxane (5 : 95%) column (PTE-5 Supelco) and fractionated in a Perkin Elmer autosystem gas chromatograph apparatus.

### 2.6. Lipid Peroxide Content Determination

Lipid peroxidation levels were assessed using the ferrous oxidation/xylenol orange (FOX) assay [19] with some modifications. Briefly, 50 mg of fresh *E. coli *Δ*yqhD* cells were suspended in 500 *μ*L of 50 mM Tris-HCl (pH 7.4) buffer, disrupted by sonication, and centrifuged at 10000 ×g for 10 min. Both crude extracts and pellets were sonicated again, this time in the presence of 1% SDS. Lipids were extracted twice with 1 volume of methanol : chloroform (1 : 2 v/v). The organic phase was rescued, evaporated in a SpeedVac, and suspended in 1 mL of the FOX reagent. After 1 h at room temperature, the lipid peroxide content was determined at 560 nm. Results were expressed as hydrogen peroxide equivalents.

### 2.7. Mass Spectrometry

One mL fractions from the HPLC aldehyde determination experiment (ranging from 15 to 17 min elution) were collected and evaporated in a Speedvac apparatus until ~10% of their original volume, pooled together and again evaporated. Samples were suspended in 50 *μ*L of methanol : H_2_O (1 : 1), and 20 *μ*L of the mixture was injected in a C18 150 × 4.6 mm column (Agilent) connected to a 1200 series chromatographer/Triple Quadrupole LC/MS Agilent 5420 with negative electrospray ionization. The LC/MS program was 0–9 min methanol : H_2_O (3 : 7), 9–16 min isocratic flux of tetrahydrofuran : H_2_O (THF : H_2_O, 26 : 74), 16–21 min isocratic flux THF : H_2_O (4 : 6), and 21–25 min isocratic flux THF 100%, nitrogen at 300°C with a flow of 6 L/min, 25 psi nebulization pressure, 4000 V of ionization, and 80–350 mass range.

## 3. Results

### 3.1. Aldehyde Generation in Stressed *E. coli*

**Figure 1:**
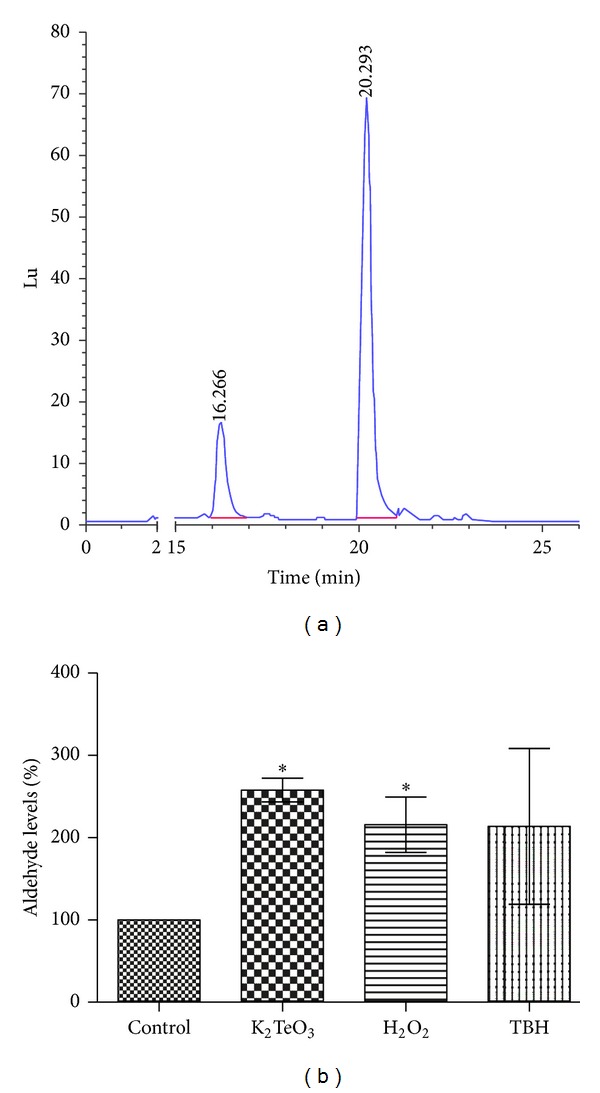
Aldehyde production in *E. coli *BW25113 Δ*yqhD*. (a) Representative chromatogram of the aldehyde eluting at ~16 min. Octanal was used as an internal standard (peak at 20 min). (b) 16 min eluting aldehyde species variation. Cells were exposed to the indicated toxicants for 30 min in minimal VB medium and used for determining aldehyde levels as described in Section 2. Error bars represent SD (*n* = 5). Asterisks indicate significant difference, *P* < 0.05.

It was previously established that *E*. *coli* lipid peroxidation levels are dependent on the membrane MUFAs ratio [13] and that peroxides derived from pure MUFA can be decomposed to aldehydes [11]. In this context, this work focused on evaluating the production of these reactive compounds in *E. coli* under oxidative stress conditions. To asses this, an aldehyde-specific HPLC fluorometric determination procedure [20] was used along with an *E. coli* strain lacking the YqhD aldehyde reductase [21] to facilitate aldehyde detection. Under normal growth conditions, an aldehyde species eluting at ~16 min (Figure 1(a)) was detected. Exposure of *E. coli* Δ*yqhD* to the oxidative stress elicitors TeO_3_^2−^, H_2_O_2_, or TBH resulted in increased aldehyde levels (Figure 1(b)). Interestingly this aldehyde was detected even in unstressed conditions (Figure 1(b), control), showing that its generation occurs in metabolically active *E. coli*.

### 3.2. Oxidative Stress Effects on the *E. coli* Membrane MUFAs

**Figure 2:**
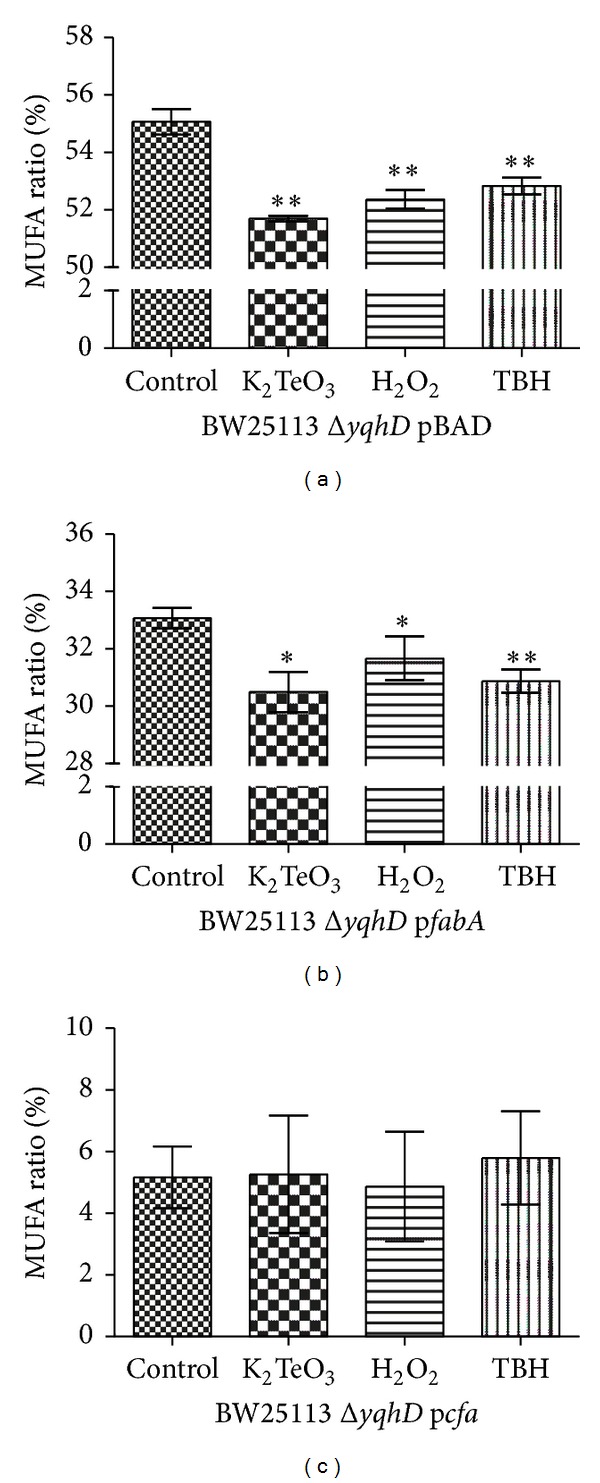
Fatty acid MUFA ratio profile (%) in *E. coli *BW25113 Δ*yqhD* exposed to the indicated toxicants. Fatty acids from *E. coli* BW25113 Δ*yqhD* carrying pBAD, p*fabA,* or p*cfa* plasmids were subjected to direct trans-esterification and analyzed by gas chromatography. Results are expressed as the percentage of total MUFA ratio in the membrane. Error bars represent SD (*n* = 5). Asterisks indicate a significant difference, *P* < 0.05.

Given the link between lipid peroxidation and aldehyde generation [10, 11] and the relationship MUFA content/lipid peroxidation previously reported [13], it is likely that MUFA → lipid peroxidation → aldehyde generation pathway takes place. To evaluate this putative relationship, the MUFA ratio (MUFA/total membrane fatty acids) was determined by gas chromatography (GC) in cells exposed or not to stress elicitors. As expected, decreased MUFA content (palmitoleic + vaccenic acid, C16 : 1 + C18 : 1) was observed under oxidative stress conditions (Figure 2(a)). 

**Figure 3:**
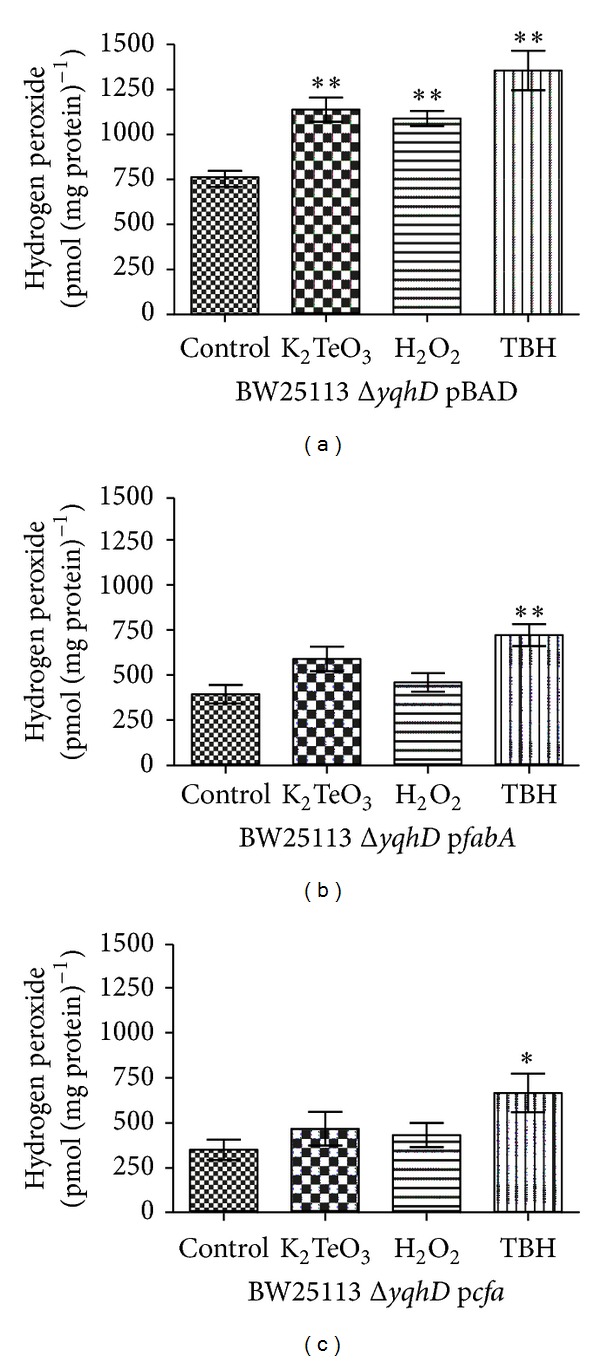
Membrane peroxide assessment in stressed *E. coli* BW25113 Δ*yqhD.* Cells carrying pBAD, p*fabA,* or p*cfa* were exposed to the indicated toxicants for 30 min in minimal VB medium and used for determining lipid peroxides as described in Section 2. Error bars represent SD (*n* = 5). Asterisks indicate significant difference, *P* < 0.05.

The fact that aldehyde levels increase while MUFAs ratio drops in ROS elicitors-exposed cells supports the idea of a link between them. In this context, lipid peroxide levels in membrane extracts from stressed *E. coli* were evaluated using the FOX assay as described in Materials and Methods. In this line and as previously stated in *E. coli* BW25113 [13], increased levels of lipid peroxidation were observed upon exposition of *E. coli *Δ*yqhD* to oxidative stress elicitors (Figure 3(a), control). These results are consistent with MUFAs being putative substrates for membrane lipid peroxidation.

### 3.3. Effect of Modulating MUFAs Ratio on Lipid Peroxidation

To further establish the link MUFA → lipid peroxidation, the effect of changing the membrane MUFA ratio in cells facing oxidative stress was evaluated. To achieve this, *fabA* and *cfa* genes, whose products are involved in the synthesis or modification of monounsaturated fatty acids [22, 23], were overexpressed in *E. coli *Δ*yqhD*. Mutants lacking these genes are unviable unless cultures are amended with the appropriate fatty acids [24]. Overexpression of *fabA* or *cfa* results in changes in the fatty acid ratio of almost all fatty acids present in the *E*. *coli *membrane. Upon *cfa* overexpression the observed decrease of the unsaturated fatty acids ratio was particularly interesting (Table 1, C16 : 1 + C18 : 1).

When cells were exposed to oxidative stress elicitors, MUFA proportions decreased (~10%) in the control, parental strain, and to a lesser extent in *fabA*-expressing *E. coli* (Figures 2(a) and 2(b)). No information could be obtained when analyzing *cfa*-expressing cells since the MUFA levels were so low that fell close to the experimental error (Figure 2(c)). 

To get a closer picture of MUFA content/aldehyde generation, lipid peroxidation levels were assessed in strains with artificially altered MUFA content. In the presence of stress elicitors, decreased lipid peroxide levels were found in strains exhibiting lower MUFA ratios (Figures 3(b) and 3(c)) than those observed in the parental strain (Figure 3(a)). 

**Figure 4:**
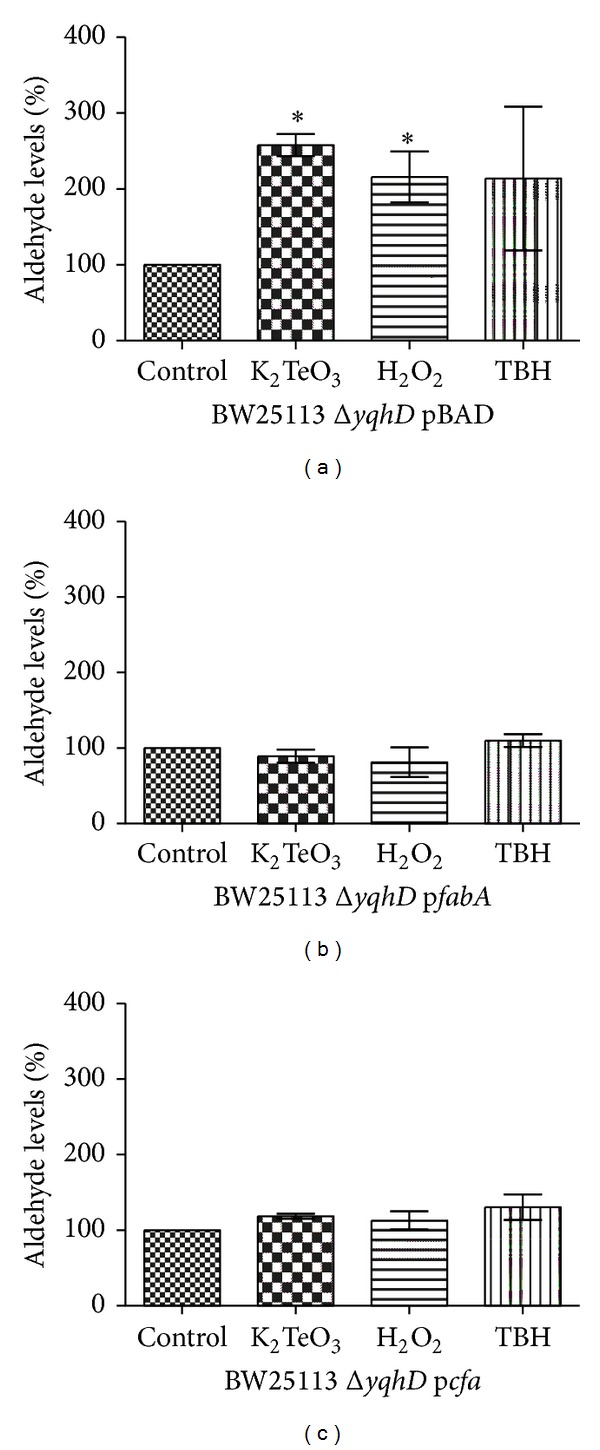
Aldehyde generation in *E. coli *exposed to oxidative stress. *E. coli* cells containing pBAD, p*fabA,* or p*cfa* were exposed to the indicated toxicants for 30 min in VB medium and used for determining aldehyde levels as described in Section 2. Error bars represent SD (*n* = 5). Asterisks indicate significant difference, *P* < 0.05.

As these results suggested a direct relationship between MUFA content and lipid peroxidation, the next step was to assess aldehyde generation in strains containing decreased MUFA ratios. As expected, in the presence of stress elicitors, cells expressing *fabA* or *cfa* (Figures 4(b) and 4(c)) showed lower aldehyde levels than the parental strain (Figure 4(a)).

### 3.4. Effects of MUFAs Modulation on Intracellular Oxidative Damage

**Figure 5:**
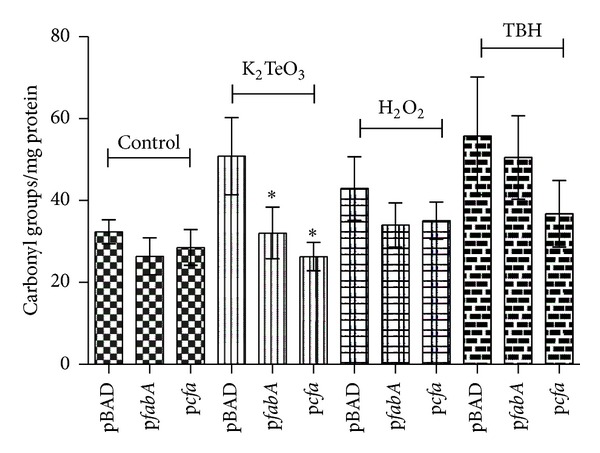
Protein carbonylation in *E. coli* exposed to different toxicants. Cells carrying pBAD, p*fabA,* or p*cfa* were exposed to the indicated oxidative stress elicitors for 30 min in VB minimal media, and carbonyl groups were assessed using 2,4 DNPH as indicated in Section 2. Error bars represent SD (*n* = 5). Asterisks indicate significant difference, *P* < 0.05.

Given that lipid peroxidation generates byproducts that may cause secondary damage to several macromolecules [25], protein carbonylation (a kind of damage normally mediated by aldehydes and other oxidative agents) [26] was assessed in stressed cells. While in the parental, untreated strain carbonyl group levels were rather unchanged, *fabA* and *cfa* over expression resulted in decreased protein oxidation mainly in tellurite-treated cells (Figure 5).

### 3.5. Effects of MUFAs Modulation on Toxicant's Minimal Inhibitory Concentrations

Since altering MUFA ratios could affect the level of ROS-mediated cell damage, the overall cell response to stress elicitors could be affected as well. In this line, resistance to the tested toxicants was assessed by determining their minimal inhibitory concentrations (MICs). When MUFA ratios decreased by 40% or 90% (p*fabA* and p*cfA*, resp.) (Table 1), tellurite resistance increased twofold. Upon *fabA* or *cfA* over expression resistance to hydrogen peroxide and TBH increased two- and fourfold, respectively, showing a link between MUFAs content and resistance to oxidative stress elicitors (Table 2). 

### 3.6. Aldehyde Identification

**Figure 6:**
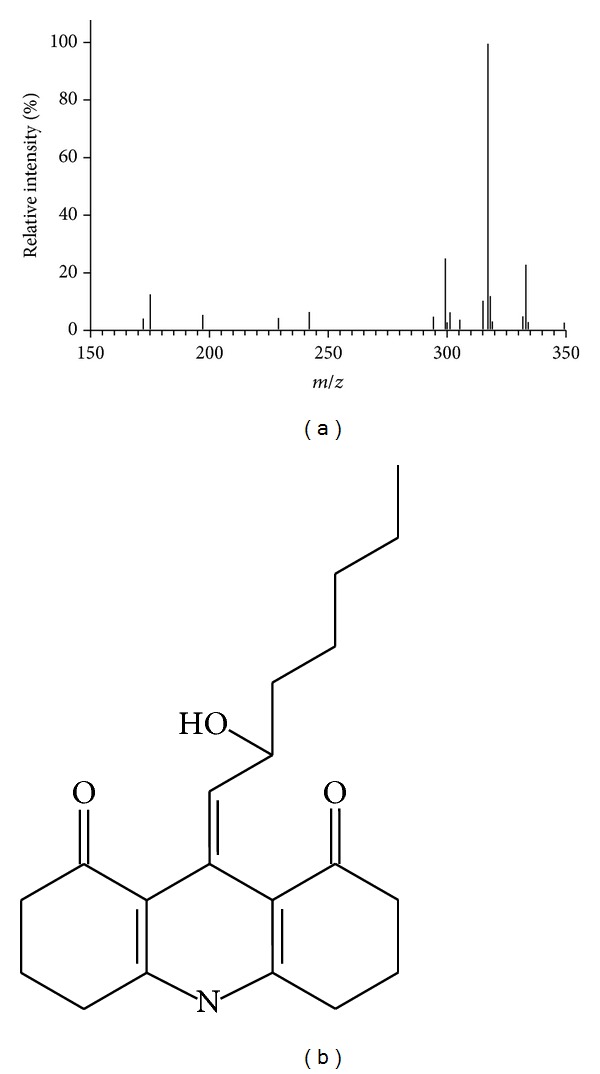
Identification of the aldehyde species by mass spectra. (a) Concentrated fractions of the *E. coli* aldehyde from the HPLC column were analyzed by LC-MS. A representative spectrum is shown (*n* = 3). (b) Determined 1,3-cyclohexanedione derivative.

To establish that the aldehyde species eluting at 16 min (Figure 1(a)) comes from MUFA-derived lipid peroxides, its identity was assessed by mass spectrometry.  Figure 6(a) shows the mass spectra for the derivatized aldehyde, whose highest *m*/*z *value was 349 (Figure 6(a)). The most reasonable fit to this spectrum corresponds to the pseudomolecular *m/z* signal expected for the 1,3 cyclohexanedione derivate [20] of 4-hydroxy 2-nonenal (Figure 6(b)).

## 4. Discussion

A previous work from our group indicated that aldehyde detoxification plays a role in resistance to oxidative stress [21]. In this context, the finding of an aldehyde species produced in unstressed conditions but whose levels increase under oxidative stress conditions (Figure 1) suggested both that its generation results from the normal, aerobic metabolism in *E. coli* [11, 13] and that it is dependent on the cell oxidative status.

Considering the proposed MUFA → lipid peroxidation → aldehyde generation pathway, the observed decrease of MUFAs levels (Figure 2(a)) and increased amounts of lipid peroxidation (Figure 3(a)) are consistent with MUFAs being substrates for membrane lipid peroxidation, with the observed augment of aldehyde levels coming most probably from lipid peroxide decomposition. Furthermore, upon MUFA ratio modulation decreased levels of lipid peroxidation (Figures 3(b) and 3(c)) and aldehyde (Figures 4(b) and 4(c)) were observed when cells were exposed to oxidative stress elicitors, supporting the idea of a direct link MUFA → lipid peroxidation → aldehyde generation. Oxidized by ROS, monounsaturated fatty acids (palmitoleic, vaccenic) would generate lipid peroxides in the same manner as oleic acid (MUFA) is oxidized to form hydroperoxides which finally decompose to reactive aldehydes [11]. Thus, the lower the MUFA content, the lower the lipid peroxidation level and aldehyde generation. 

On the other hand, one noteworthy aspect of the MUFA → lipid peroxidation → aldehyde generation link is represented by the impact of MUFA ratio modulation on oxidative damage within the cell, particularly by cell exposition to tellurite (Figure 5). This result is intriguing since previous findings indicated that the observed effects were independent of the toxicant. In this context and given that peroxides pass freely through the cell membrane [6], it is conceivable that shortly after toxicant exposure they could affect all cell components rather than exclusively the membrane. This may possibly make peroxide-mediated protein carbonylation less dependent on products derived from membrane oxidation. On the other hand, when compared to TBH and H_2_O_2_, the intimate interaction of tellurite with the *E. coli* membrane [27] could result in a localized effect that may enhance any damage derived from membrane oxidation byproducts. These results suggest that the* E. coli* membrane composition is relevant regarding the extent of cell damage caused by oxidative stress elicitors. Similar results of damage by membrane-derived byproducts such as peroxides and/or aldehydes have been observed in other organisms [8, 28]. 

Given the observed inverse relationship between MUFA content and resistance to ROS elicitors (Table 2), the relevance of the *E. coli* membrane composition also seems to be relevant to the bacterial resistance towards oxidative stress. Interestingly, cells exposed to NiCl_2_, a toxicant that does not produce oxidative damage, exhibited no differences in resistance levels compared with untreated controls (data not shown). These results are in agreement with those of Pesakhov et al. [29], who observed enhanced resistance to oxidative stress upon MUFA depletion from the *E. coli* membrane. Due to the potential effects of MUFA ratio alterations on membrane fluidity, we cannot fully disregard a variation in toxicant access through the membrane. However this seems rather unlikely because NiCl_2_ toxicity is not affected by MUFA ratio variations. Since lipid peroxide and aldehyde detoxification systems seem to be part of the *E. coli* response to oxidative stress elicitors [5, 30, 31], the observed increase in resistance to these agents could be more likely related to decreased lipid peroxide and aldehyde levels. 

Finally, the identity of the detected aldehyde also suggests an MUFA-derived origin for this compound. Based on the published models for aldehyde generation from oleic acid [11], a mechanism that could explain the generation of 4-hydroxy 2-nonenal is proposed; this aldehyde might derive from the autooxidation of 10- and/or 13-vaccenic acid hydroperoxide which in turn could come from vaccenic acid oxidation (see Figure S1 in the Supplementary Material available online at http://dx.doi.org/10.1155/2013/563756). This model is in agreement with the results showing decreased MUFA ratios in *fabA*- and *cfa*-expressing cells, where a preferential decrease of the vaccenic acid content was observed (Table 1). The autooxidation step in the proposed model is supported by former evidence indicating that aerobic conditions are required for aldehyde production [21] and for autooxidation to occur [32].

## 5. Conclusion

Our results show that oxidative stress elicitors affect the *E. coli* membrane MUFA ratio. The production of an aldehyde derived from these fatty acids—most likely 4-hydroxy 2-nonenal—increases upon toxicant exposure. Finally, a direct relationship between membrane MUFAs and the generation of lipid peroxides and reactive aldehydes was found, which seems to modulate—at least in part—the cell's tolerance to oxidative stress elicitors.

## Supplementary Material

Supplementary Figure 1: Vaccenic acid-derived aldehyde formation. Mechanistic explanation for the generation of 4-hydroxy 2-nonenal. The asterisks show activated sites.

## Figures and Tables

**Table 1:** Fatty acid ratio in * E. coli* carrying pBAD, p*fabA*, or p*cfA* plasmids.

Strain/plasmid	Fatty acid ratio (%)					
BW25113 Δ*yqhD *	C16 : 1	C16	C17	C18 : 1	C19	C16 : 1 + C18 : 1
pBAD	18.2 ± 0.5	35.3 ± 0.5	10 ± 0.8	36.9 ± 0.9	0	55.2 ± 0.5
p*fabA *	16.6 ± 1.0	59.4 ± 1.0	7.4 ± 1.4	16.5 ± 0.7	0	33.1 ± 0.4
p*cfa *	1.7 ± 0.5	27.7 ± 0.7	29.3 ± 1.1	3.6 ± 0.6	37.7 ± 0.8	5.3 ± 1.0

Total fatty acids from the indicated * E. coli* strains were subjected to direct transesterification and analyzed by gas chromatography. Results are expressed as the percentage of total fatty acids. C16 :  1, palmitoleic acid; C16, palmitic acid; C17: margaric acid; C18 : 1, vaccenic acid; C16 : 1 + C18 : 1, and variation of total MUFA content (C16 : 1 + C18 : 1); C19: nonadecylic acid.

**Table 2:** MIC of the indicated toxicants for *E. coli* carrying pBAD, p*fabA*, or p*cfa*.

*E. coli* BW25113 Δ*yqhD *	K_2_TeO_3_ (*µ*M)	H_2_O_2_ (*µ*M)	TBH (*µ*M)
pBAD	1	1250	60
p*fabA *	2	2500	250
p*cfa *	2	2500	250
