# Supplementary figures and images for: Monounsaturated Fatty Acids Are Substrates for Aldehyde Generation in Tellurite-Exposed Escherichia coli
Source: Biomed Res Int. 2013 Aug 7;2013:563756. doi: 10.1155/2013/563756 (PMC3749545; doi:10.1155/2013/563756)

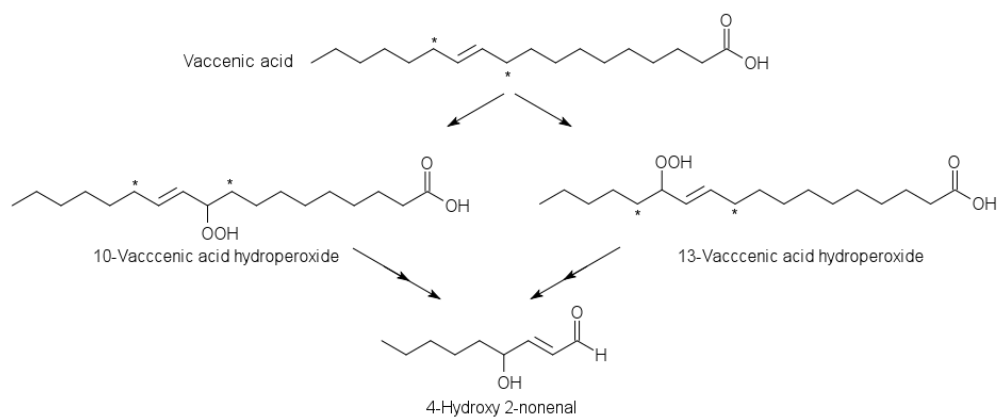

**Fig. S1.** Pradenas *et al.*

Supplement: Supplementary file 1 — Supplementary Figure 1: Vaccenic acid-derived aldehyde formation. Mechanistic explanation for the generation of 4-hydroxy 2-nonenal. The asterisks show activated sites. [file 563756.f1.pdf]
